# F256. CAN WE REDUCE THE DURATION OF UNTREATED PSYCHOSIS?

**DOI:** 10.1093/schbul/sby017.787

**Published:** 2018-04-01

**Authors:** Dominic Oliver, Cathy Davies, Georgia Crossland, Steffiany Lim, George Gifford, Philip McGuire, Paolo Fusar-Poli

**Affiliations:** Institute of Psychiatry Psychology and Neuroscience, King’s College London

## Abstract

**Background:**

Reduction of duration of untreated psychosis (DUP) is the key strategy of early interventions for improving the outcomes of first episode psychosis. Although several controlled interventional studies have been conducted with the aim of reducing DUP, the results are highly inconsistent and conflicting.

**Methods:**

The current study systematically searched Web of Science and Ovid for English original articles investigating interventions adopted to reduce DUP, compared to a control intervention, up to 6th April 2017. 16 controlled interventional studies were retrieved, including 1964 patients in the intervention arm and 1358 in the control arm. The controlled interventions studies were characterised by: standalone first episode psychosis services, standalone clinical high risk services, community interventions, healthcare professional training and multifocus interventions. Random effects meta-analyses were conducted.

**Results:**

There was no summary evidence that available interventions are successful in reducing DUP during the first episode of psychosis (Hedges’ g = -0.12, 95%CIs -0.25 to 0.01). Subgroup analyses showed no differences within each subgroup, with the exception of clinical high risk services (Hedges’ g = -0.386, 95%CI -0.726 to -0.045). . There was substantial heterogeneity (I2 = 66.4%), most of which was accounted by different definitions of DUP onset (R2=0.88).

**Discussion:**

These negative findings may reflect a parcelled research base in the area, lack of prospective randomized controlled trials (only two randomised cluster designed studies were present) and small sample sizes. Psychometric standardisation of DUP definition, improvement of study design and implementation of preventative strategies seem the most promising avenues for reducing DUP and improving outcomes of first-episode psychosis.

